# YAP Inhibition by Nuciferine via AMPK-Mediated Downregulation of HMGCR Sensitizes Pancreatic Cancer Cells to Gemcitabine

**DOI:** 10.3390/biom9100620

**Published:** 2019-10-17

**Authors:** Ling Zhou, Qiaoyun Wang, Han Zhang, Youjie Li, Shuyang Xie, Maolei Xu

**Affiliations:** 1 The Key Laboratory of Traditional Chinese Medicine Prescription Effect and Clinical Evaluation of State Administration of Traditional Chinese Medicine, School of Pharmacy, Binzhou Medical University, Yantai 264003, China; zhouling-2007-ok@163.com (L.Z.); byylwqy@163.com (Q.W.);; 2 Key Laboratory of Tumor Molecular Biology in Binzhou Medical University, Department of Biochemistry and Molecular Biology, Binzhou Medical University, Yantai 264003, China; youjie1979@163.com

**Keywords:** Nuciferine, Yes-associated protein, AMP-activation protein kinase, 3-hydroxy-3-methyl-glutaryl-coA reductase, gemcitabine, pancreatic cancer

## Abstract

Nuciferine, a major aporphine alkaloid constituent of lotus leaves, is a raw material for obesity treatment. Extensive studies have revealed that obesity is associated with pancreatic cancer (PC). However, it has not been clarified whether nuciferine could be used in PC treatment or prevention. Here, we show that nuciferine could enhance the sensitivity of PC cells to gemcitabine in both cultured cells and the xenograft mouse model. The mechanism study demonstrated that nuciferine induced YAP Ser127 phosphorylation [pYAP(Ser127)] through AMPK-mediated 3-hydroxy-3-methyl-glutaryl-coA reductase (HMGCR) downregulation. Remarkably, wild-type YAP overexpression or YAP Ser127 mutant could resist to nuciferine and no longer sensitize PC cells to gemcitabine. Knockdown of AMPK attenuated pYAP(Ser127) induced by nuciferine. Moreover, knockdown of AMPK reversed nuciferine-mediated HMGCR downregulation. Notably, HMGCR inhibiting could restrain YAP by phosphorylation Ser 127, and therefore enhance the efficiency of gemcitabine in PC cells. In line with this consistent, overexpression of HMGCR reduced growth inhibition caused by nuciferine and/or gemcitabine treatment in PC cells. In summary, these results provide an effective supplementary agent and suggest a therapeutic strategy to reduce gemcitabine resistance in PC.

## 1. Introduction

Pancreatic cancer (PC) is the seventh leading cause of cancer-related deaths worldwide. Since patients seldom exhibit symptoms until an advanced stage of the disease, PC remains one of the most lethal malignant neoplasms that caused 432,242 new deaths in 2018 worldwide (GLOBOCAN 2018 estimates). Worldwide incidence and mortality of PC correlate with increasing age and is slightly more common in men than in women [1]. Despite the great progress achieved in the last couple of decades, surgical intervention still remains the optimal treatment option. However, due to the late diagnosis and early metastasis, patients with PC usually lose the opportunity to undergo radical surgery. Therefore, identifying new drugs or therapeutic treatments to address this troublesome disease is urgently needed.

AMP-activation protein kinase (AMPK) is an energy sensor and master regulator of metabolism. Several studies have revealed that AMPK is closely related to drug resistance [2,3]. There are some small molecular inhibitors and bioactive compounds extracted from natural products improving multidrug resistance through activation of AMPK [4,5]. Additionally, the combination between AMPK activators and chemotherapeutics demonstrates good results on tumour growth inhibition and prolongs remission of breast, pancreatic, prostate, lung and ovarian cancers [6,7,8,9,10].

YES-associated protein (YAP), which is the major downstream effector of the Hippo pathway is dysregulated in PC [11]. Several studies have indicated that YAP could promote PC development and progression [12,13]. Given the essential of cellular energy and metabolites for survival and growth, YAP is inhibited when AMPK is activated. Indeed, accumulating evidence has verified that AMPK is a negative regulator of YAP activity. Activation of AMPK induces an increase in YAP phosphorylation, favors YAP cytoplasm localization, and inhibits YAP target genes [14,15,16]. However, there are several mechanisms by which this occurs. One mechanism is that AMPK directly binds to YAP and phosphorylates it on Ser61 and Ser94 [14], which reduces target gene expression and impair YAP- TEA domain (TEAD) interaction. Another mechanism, which AMPK could reduce YAP activity is to phosphorylate angiomotin-like 1 (AMOTL1) and promote LATS1 activity by activated AMPK and then facilitates the phosphorylation of YAP by LATS1 on Ser 127 [15], thereby excluding it from the nucleus. A third finding indicates that RHO regulates YAP in a manner that is independent of the LATS1/2 Hippo pathway kinases [16].

Gemcitabine is a standard first line chemotherapeutic agent for patients with advanced PC. However, PC chemotherapy is frequently impeded by gemcitabine resistance. Combination anticancer drugs with different modes of action don’t avoid the development of gemcitabine resistance. Therefore, identifying supplementary agents that sensitize PC cells to gemcitabine and help to attenuate drug resistance would be valuable for improving patient tolerance and response to chemotherapy. Natural products, especially those from medicinal and food plants, have displayed potent cancer chemopreventive and chemotherapeutic activity in both preclinical and clinical studies [17,18]. Nuciferine, a major aporphine alkaloid extracted from lotus leaves, is a raw material in chinese medicinal herb for obesity treatment. Extensive studies have revealed that obesity is associated with PC [19,20]. However, it has not been clarified whether nuciferine could be used in PC treatment or prevention. Evidences have proved that dysregulation of YAP could induce chemoresistance [21,22,23]. In this study, we explored the involvement and mechanism of YAP in nuciferine attenuating gemcitabine resistance of PC, with the aim of finding a potential supplementary agent. Our results showed that nuciferine efficiently inhibits YAP and enhances the sensitivity of PC to gemcitabine through AMPK-mediated downregulation of 3-hydroxy-3-methyl-glutaryl-coA (HMGCR). These findings provide an evidence that nuciferine is a supplementary agent to reduce gemcitabine resistance in PC.

## 2. Materials and Methods

### 2.1. Antibodies and Reagents

Anti-AMPKα (no. 2532), pAMPKα (Thr 172) (no. 2535), YAP (no. 4912), pYAP (Ser 127) (no. 4911), LATS1 (no. 3477), LATS2 (no. 5888), pLATS1 (Ser 909) (no. 9157) and pLATS1 (Thr 1079) (no. 8654) were obtained from Cell Signaling. Anti-AMOTL1 (ab171976), HMGCR (ab174830) and β-actin (ab8227) were obtained from Abcam. Anti-pYAP(Ser61) and p-YAP(Ser94) were obtained from ABclonal (Wunhan, China). Nuciferine (≥98% purity) was purchased from Nature Standard Technical Service co., Ltd. (Shanghai, China). Nuciferine was dissolved in Dimethyl Sulfoxide (DMSO) then made into the storage solution with the concentration of 0.1 M. Working dilutions for nuciferine were prepared in culture medium, and DMSO was used as control. Gemcitabine was purchased from Eli Lilly (Indianapolis, IN, USA) and dissolved in sterile phosphate buffered solution (PBS) before use. MTT was obtained from Fluka Chemical Corp. (Ronkonkoma, NY, USA) and was dissolved in 0.01 M PBS.

### 2.2. Cell Lines and Cell Culture

The human PC cell lines PANC-1, BxPC-3 and ASPC-1 were purchased from Cell Bank of Type Culture Collection of the Chinese Academy of Sciences (Shanghai, China). PANC-1 and ASPC-1 were maintained in DMEM media and BxPC-3 were cultivated in 1640 media (GIBCO, NY, USA). All media contained 10% heat-inactivated fetal bovine serum (FBS, GIBCO, USA), 100 U/mL penicillin and 100 μg/mL streptomycin (GIBCO, USA). All cell lines were incubated 37 °C in a 5% CO_2_ atmosphere.

### 2.3. MTT Assay

Cell viability was determined by 3-(4 5-Dimethylthiazol-2-yl)-2 5-diphenyltetrazolium bromide (MTT) assay. PANC-1, BxPC-3 and ASPC-1 cells were treated with nuciferine and/or gemcitabine for 72 h at various concentrations. The formazan was dissolved in DMSO and the absorbance was measured by the Universal Microplate Reader (BIO-TEK instruments, Inc., Vermont, MA, USA). The cell viability was calculated by the following formula: (A treated/A control) × 100%. Drug interaction between nuciferine and gemcitabine was assessed using the combination index (CI), and CI was calculated using the Chou-Talalay equation [24].

### 2.4. Colony Formation Assay

PANC-1 cells were plated (200,000 per well) in a six-well plate and incubated overnight. After nuciferine and/or gemcitabine treatment for 24 h, cells were trypsinized and viable cells were counted. Then 1000 viable cells were plated in 100-mm Petri dishes and incubated for 14 days. Colonies were fixed using 4% paraformaldehyde and then stained for 15min using 2% crystal violet. The number of colonies was counted as described previously [25]. 

### 2.5. Western Blot Assay

Transplanted tumor tissue or cell protein were extracted with lysis buffer. The concentration of protein was determined by BCA assay kit (Beyotime, Shanghai, China). Protein samples were analyzed by SDS-PAGE using a 10%–12% polyacrylamide and transferred onto the NC membranes (Millipore, Billerica, MA, USA). Immune complexes were formed by incubation of primary antibodies for overnight at 4 °C, then followed conjugated second antibody for 1 h at 37 °C. Immunoreactive protein bands were detected with Tanton chemiluminescenc image analysis system (Shanghai, China), and the density of protein band was detected by Image J software.

### 2.6. Real-Time PCR Analysis

After nuciferine treatment for 24 h, PANC-1 cells were lysised using the Trizol Reagent (Thermo Fisher Scientific, Waltham, MA, USA) and total RNA was extracted. cDNAs were synthesized and RT-PCR was performed on the CFX96™ Real-Time PCR Detection System (BIO-RAD, Hercules, CA, USA). The primer sets used in the PCR assay were as follows:

β-actin- Forward (5′-TCCTTCCTGGGCATGGAGTC-3′),
β-actin- Reverse (5′-TTCTGCATCCTGTCGGCAATG-3′);
CYR61- Forward (5′-AGCCTCGCATCCTATACAACC-3′),
CYR61- Reverse (5′-GAGTGCCGCCTTGTGAAAGAA-3′);
CTGF- Forward (5′-CCAATGACAACGCCTCCTG-3′),
CTGF- Reverse (5′-GAGCTTTCTGGCTGCACCA-3′).

### 2.7. ATP and ADP Quantification Assays

Cellular ATP and ADP quantification were measured using ADP/ATP Ratio Assay Kit (Sigma, St Louis, MO, USA). PANC-1 and ASPC-1 cells plated in 12-well plates were incubated with or without nuciferine for 72 h, and the cultured cells were lysed to release ATP and ADP. ATP and ADP level were determined according to the manufacturer’s instructions. 

### 2.8. Immunoprecipitation

PANC-1 cells were treated with nuciferine (50 μM) for 24 h. After treatment, the cells were washed and lysed for 15 min on ice and then centrifuged at 12000× *g* for 10 min and the soluble fraction was collected. Immunoprecipitation analysis AMPK was immunocaptured from total cell extracts using antibodies to AMPK crosslinked to protein A-agarose beads (Santa Cruz, CA, USA). The complexes were analyzed by Western blot and detected with antibody against YAP.

### 2.9. Immunofluorescent (IF) Staining

After treatment with nuciferine (50 μM) for 24 h, PANC-1 cells were washed with cold PBS, fixed with 4% paraformaldehyde for 20 min and permeabilized with 0.2% Triton X-100 for 5 min. After incubated with 5% BSA for 1 h, cells were incubated with anti-YAP antibody overnight at 4 °C. After being washed twice, the cells were incubated with FITC-labeled goat anti-rabbit IgG (H+L) antibody (Jackson ImmunoResearch, PA, USA) for 1 h at 37 °C. In addition, the coverslips were stained with DAPI for 15 min. The images were captured with a confocal scanning microscope (ZEISS LSM800, Jena, Germany).

### 2.10. Small Interfering RNA (siRNA) Transient Transfection

siRNA targeting AMPK, LATS1/2 and HMGCR for knockdown were purchased from GenePharma (Shanghai, China). The siRNAs were delivered using Lipofectamine 3000 (Invitrogen Life Technologies, CA, USA) according to previous researche [26]. After formation of the siRNA-liposome complexes, the mixture was added to cells for 6 h.

### 2.11. Plasmid Extraction and Transfection

The wild-type YAP plasmid, mutant YAP(c.781T→G, encoding p.Ser127Ala) plasmid and HMGCR plasmid were synthesized by Genechem (Shanghai, China). EndoFree Plasmid Midi Kit from Beyotime (Shanghai, China) was chosen for plasmids extraction. For transfection, PANC-1 cells were seeded in 6-well plates at 65% confluency. Then, the plasmid DNA was introduced into the cells using Lipofectamine 3000 following the instructions.

### 2.12. Animal Tumor Model and Treatments

5-weeks-old female BALB/c nude mice were obtained from Jinan Peng Yue experimental animal breeding Co. Ltd. (Jinan, Shandong, China, permission number: SCXK(LU)20140007). All experiments on animals were complied with the Binzhou Medical University’s Policy on the Care and Use of Laboratory Animals. PANC-1 cells (1 × 10^7^) were injected subcutaneously into the right flank of mice. After 4 weeks, nude mice with the xenograft tumour sizes of approximately 100 mm^3^ were randomly assigned to four groups (n = 4, each group): Vehicle group, Nuc treatment (intraperitoneally [IP] injected with Nuc at a dose of 30 mg/kg, once/every other day), Gem treatment (20 mg/kg by IP injection twice weekly) and the combination treatment of Nuc and Gem (30 mg/kg Nuc once/every other day and 20 mg/kg gemcitabine twice weekly). Tumor volume and mice body weight were measured every three days. The tumor volume was calculated using the formula, V = length × width^2^/2. After therapy was continued for 4 weeks, mice were sacrificed and tumor samples were excised and weighed. The major organ sections were excised for toxic evaluation.

### 2.13. Histological Analysis

The major organ (heart, liver, spleen lung and kindy) sections were fixed in 4% paraformaldehyde solution, and then embedded and sectioned for Hematoxylin-eosin staining. Images were captured using a light microscope (Leica DM6000B, Munich, Germany).

### 2.14. Statistical Analysis

Each experiment was repeated three times, unless otherwise indicated. Data were presented as mean ± SD from triplicate parallel experiments. Statistical analysis were performed using one-way ANOVA.

## 3. Results

### 3.1. Nuciferine Attenuates Gemcitabine Resistance of Pancreatic Cancer Cells

**Figure 1:**
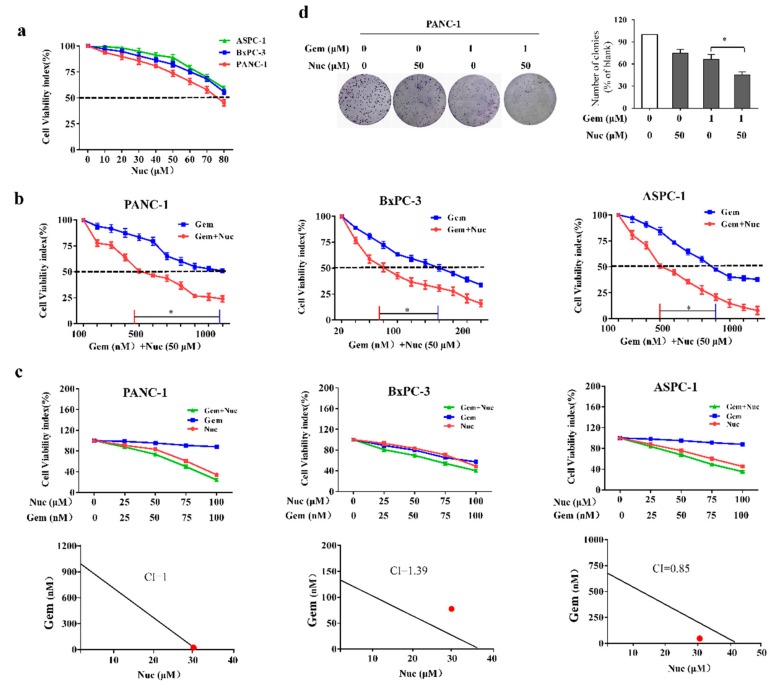
Nuciferine sensitizes pancreatic cancer cells to gemcitabine. (**a**) Effects of nuciferine on the proliferation of PANC-1, BxPC-3 and ASPC-1 cells. (**b**) Viability of three pancreatic cancer cell lines treated with different concentrations of gemcitabine in the absence or presence of nuciferine (50 μM) for 72 h. (**c**) Drug interaction between nuciferine and gemcitabine was assessed using the combination index (CI). (**d**) Clonogenicity of PANC-1 cells following treatment with nuciferine and/or gemcitabine for 24 h. Nuc, nuciferine; Gem, gemcitabine. * *p* < 0.05.

We first examined the abilities of nuciferine to suppress tumor growth in PANC-1, BxPC-3 and ASPC-1 cell lines. As observed in Figure 1a, treatment with nuciferine by itself with concentrations up to 50 μM didn’t elicit optimal growth inhibitory effects on PC cells as IC_50_ values were not obtained. To determine whether nuciferine could enhance the susceptibility of PC cells to gemcitabine, combination treatments were carried out by varying gemcitabine in the presence of nuciferine for 72 h. As shown in Figure 1b, addition of a suboptimal dose of nuciferine lowered the gemcitabine IC_50_ from 1120 nM to 402 nM (2.8-fold) in PANC-1 cells, 164 nM to 66 nM (2.48 fold) in BxPC-3 cells and 720 nM to 305 nM (2.36 fold) in ASPC-1 cells. These experiments demonstrated that suboptimal dose of nuciferine could decrease the doses of gemcitabine required to reach the IC_50_ for these PC cell lines. Moreover, these experiments showed the differential sensitivity of these three PC lines to gemcitabine, almost a 7-fold difference in sensitivity between PANC-1 cells and BxPC-3 cells. Furthermore, the Chou-Talalay CI indicated that gemcitabine and nuciferine synergistically enhanced cytotoxicity in ASPC-1 cells (CI = 0.85; Figure 1c). On the other hand, the combined treatment of gemcitabine and nuciferine enhanced cellular cytotoxicity additively in PANC-1 cells (CI = 1; Figure 1c). Althrough combination therapy did further enhance cellular cytotoxicity, the CI showed no synergism and additive effect between nuciferine and gemcitabine in BxPC3 cells (CI = 1.39; Figure 1c).

Next, the antiproliferative activity of nuciferine and/or gemcitabine was further evaluated by colony formation assay. In PANC-1 cells, treatment with either compound effectively reduced colony number compared with controls, and the combination of nuciferine and gemcitabine further inhibited the clonogenic capacity (*p* < 0.05, Figure 1d). Taken together, these results suggest that nuciferine was not only cytotoxic to PC cells but also sensitized PC cells to gemcitabine and suppressed cellular proliferation.

### 3.2. Nuciferine Activates AMPK in Pancreatic Cancer Cells

**Figure 2:**
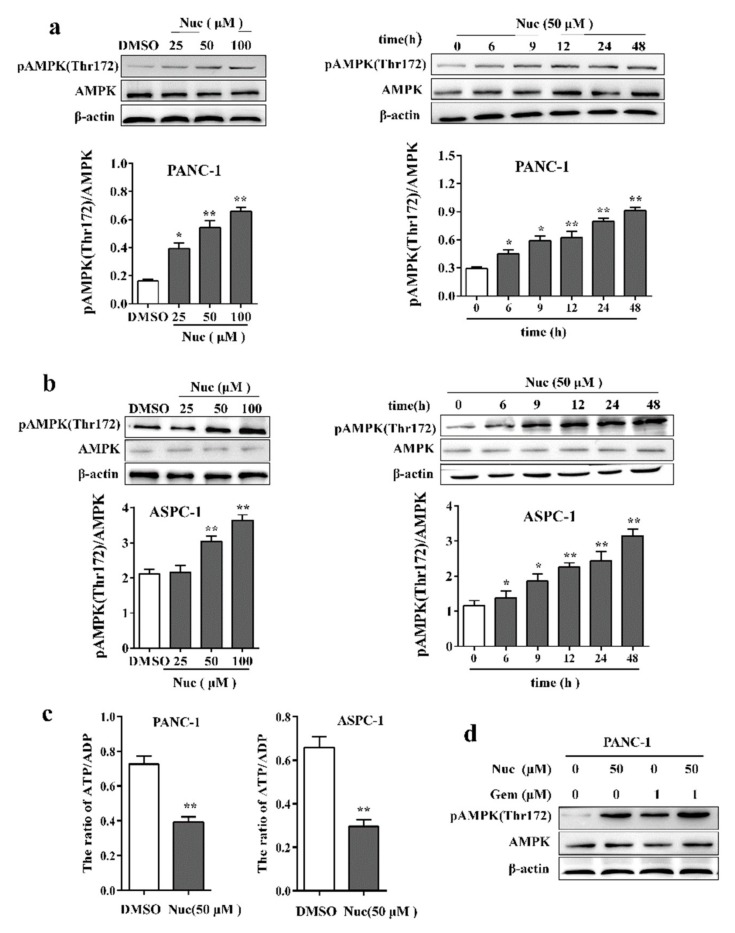
Nuciferine activates AMPK in PANC-1 and ASPC-1 cells. (**a**,**b**) The whole-cell lysates were prepared and subjected to western blot for pAMPK(Thr172) and AMPK, and relative density between AMPK and pAMPK(Thr172) was determined. (**c**) Levels of ATP and ADP were detected and the ratio of ATP to ADP was calculated. (**d**) After treatment with nuciferine and/or gemcitabine, protein level of AMPK and pAMPK(Thr172) was determined. Nuc, nuciferine; Gem, gemcitabine. * *p* < 0.05 or ** *p* < 0.01 versus DMSO-treated control cells.

A growing body of evidence has suggested that activation of AMPK is pivotal to increase the susceptibilities of tumor cells to chemotherapy drugs [27,28]. To determine whether AMPK activation is susceptible to nuciferine enhanced the sensitivity of PC cells to gemcitabine, we examined the effect of nuciferine on the activation of AMPK in PC cells. A dose-dependent study in PANC-1 cells revealed a moderate increase in phosphorylation of AMPK at Thr 172, the active form of AMPK, 48 h after exposure to 50 μM nuciferine and very extensive increase at concentrations of 100 μM (Figure 2a). Time-course analysis of cells exposed to 50 μM nuciferine demonstrated a significant increase in pAMPK (Thr172) as early as 6 h. These events became apparent after 12 h of nuciferine exposure, and reached near maximal levels after 24 h (Figure 2a). To determine whether nuciferine mediated AMPK activation observed in PANC-1 cells also occur in other PC cell lines, parallel studies were carried out in ASPC-1 cells. Also, these cells exhibited comparable degrees of AMPK activation (Figure 2b). Moreover, we measured ATP/ADP ratios in nuciferine –treated PC cells. Our results showed that the nuciferine treatment significantly decreased ATP/ADP ratio (Figure 2c), which is the indicator of activation of AMPK. Notably, treatment combining nuciferine and gemcitabine induced more pAMPK (Thr172) than monotherapy (Figure 2d), suggesting that AMPK activation was involved in nuciferine enhanced the sensitivity of PC cells to gemcitabine.

### 3.3. Nuciferine Induces YAP Ser127 Phosphorylation and Cytoplasmic Retention

**Figure 3:**
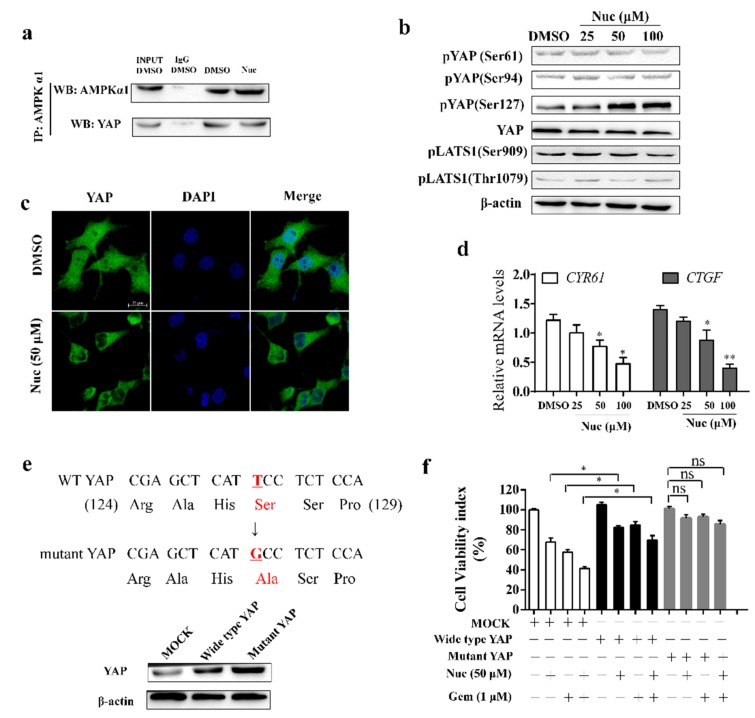
Nuciferine induces YAP Ser127 phosphorylation and inactivation. (**a**) Dimethyl Sulfoxide (DMSO-) or nuciferine -treated cells were lysed and AMPKα1 was immunoprecipitated. Western blot assays were performed for AMPKα1 and YAP. (**b**) The whole-cell lysates were prepared and subjected to western blot for pYAP(Ser61), pYAP(Ser94), pYAP(Ser127), YAP, pLATS1(Ser909) and pLATS1(Thr1079). (**c**) YAP nuclear translocation in PANC-1 cells stimulated with nuciferine (50 μM) for 24 h. Scale bars: 20 μM. (**d**) Real-time quantitative PCR of CYR61 and CTGF. * *p* < 0.05 or ** *p* < 0.01 versus DMSO-treated cells. (**e**) Protein expression of YAP in PANC-1 cells transfected with wild-type YAP or mutant YAP (c.781T→G, encoding p.Ser127Ala). (**f**) Cells transfected with wild-type YAP or mutant YAP overexpressed plasmid were treated with nuciferine and/or gemcitabine for 72 h and cell viability was determined. Data were showed as mean ± SD. Nuc, nuciferine; Gem, gemcitabine; WT, wide type. * *p* < 0.05 and ns = not significant.

AMPK has been shown to oppose the growth-promoting activity of YAP, which promote chemoresistance in PC cells. To examine the role of YAP inhibition in nuciferine-elevated efficiency of gemcitabine, we examined the effect of nuciferine on combination of AMPK protein and YAP. As co-immunoprecipitation assays showed that nuciferine didn’t enhance the combination of AMPK kinase subunits, which were identified as YAP-associated proteins, and YAP (Figure 3a). Concordantly, nuciferine treatment didn’t increase YAP S61 or S94 phosphorylation (Figure 3b). Activation of AMPK induces an increase in YAP phosphorylation at Ser127 and favors YAP localization to the cytoplasm. As shown in Figure 3b, nuciferine treatment markedly increased YAP Ser 127 phosphorylation, while phosphorylation of Ser 909 and Thr 1079 in LATS1 which is positively correlates with LATS activity [29,30] has no noticeable change. The confocal microscopic image showed that nuciferine treatment decreased nuclear YAP staining (Figure 3c). In this regard, the downstream target genes of YAP (CYR61 and CTGF) were significantly downregulated in nuciferine treated PANC-1 cells (Figure 3d). Thus, nuciferine impair the nuclear translocation of YAP acts through inducing YAP Ser127 phosphorylation.

We next explored whether overexpression of wild-type YAP or YAP Ser127 mutant could resist to nuciferine and no longer sensitized PC cells to gemcitabine. When PANC-1 cells were transfected with wild-type YAP plasmid, cell viability in cells with nuciferine and/or gemcitabine treatment was further increased (Figure 3e,f), and transfection of YAP mutant plasmid completely abolished cytotoxicity induced by nuciferine, gemcitabine or their combination (Figure 3e,f). These results further confirmed that nuciferine enhanced the efficacy of gemcitabine through inducing pYAP(Ser127).

### 3.4. Nuciferine Inhibits YAP Involving AMPK-Mediated Downregulation of HMGCR

**Figure 4:**
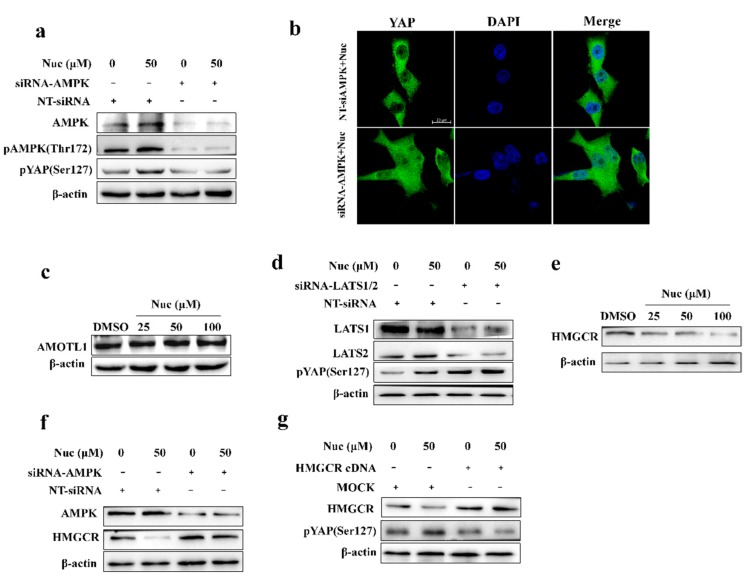
AMPK-mediated 3-hydroxy-3-methyl-glutaryl-coA (HMGCR) downregulation is needed for nuciferine to induce pYAP(Ser127). (**a**) Western blot of AMPK, pAMPK(Thr172), pYAP(Ser127) and β-actin from PANC-1 cells transfected with none-targeted siRNA(NT-siRNA) and siRNA-AMPK. (**b**) YAP nuclear translocation in PANC-1 cells transfected with NT-siRNA and siRNA-AMPK in the presence of nuciferine (50 μM). Scale bars: 20 μm. (**c**) Western blot of AMOTL1 and β-actin (a loading control); (**d**) Western blot of LATS1, LATS2, pYAP(Ser127) and β-actin from PANC-1 cells transfected with NT-siRNA and siRNA-LATS1/2. (**e**) Cells were treated with nuciferine or DMSO for 72 h and collected for WB. (**f**) Western blot of AMPK and HMGCR from cells transfected with NT-siRNA and siRNA-AMPK. (**g**) Cells transfected with HMGCR overexpressed plasmid were treated with nuciferine or DMSO for 72 h and collected for western blot. Nuc, nuciferine; Gem, gemcitabine.

To investigate the role of AMPK in nuciferine-regulated YAP activity, we used siRNAs targeting AMPKa1 and AMPKa2 to knock down AMPK in PANC-1 cells. The siRNAs greatly inhibited pYAP(Ser127) induced by nuciferine, suggesting that AMPK is required to nuciferine-mediated the regulation of YAP (Figure 4a). Consistently, we also observed that AMPK knockdown rescued the effects of nuciferine on YAP nuclear localization (Figure 4b). In addition, nuciferine treatment didn’t increase the protein level of AMOTL1 (Figure 4c) which become more stable after phosphorylated by AMPK, thereby promoting the phosphorylation of YAP by LATS on serine 127. Furthermore, knockdown of LATS1/2 didn’t attenuate nuciferine-mediated YAP phosphorylation (Figure 4d). These results further indicate that nuciferine induce YAP phosphorylation through AMPK-mediated LATS-independent manner.

We then set out to study how nuciferine regulates YAP activity. Previous studies suggested that RHO regulates YAP is largely independent of the LATS1/2 Hippo pathway kinases and mevalonate cascade is required for activation of RHO [16,31]. Therefore, we investigated whether nuciferine-mediated the inhibition of YAP is associated with the down-regulation of HMGCR, the major regulator of the mevalonate pathway. Western blot analysis showed that nuciferine treatment decreased the protein level of HMGCR in PC cells (Figure 4e). Moreover, knockdown of AMPK reversed nuciferine-mediated HMGCR downregulation (Figure 4f). Notably, the HMGCR overexpression obviously attenuated YAP phosphorylation (Figure 4g). These results suggest that nuciferine may inhibit YAP by activating the AMPK-HMGCR axis.

### 3.5. Nuciferine Sensitizes Pancreatic Cancer Cells to Gemcitabine by Down-Regulating HMGCR

**Figure 5:**
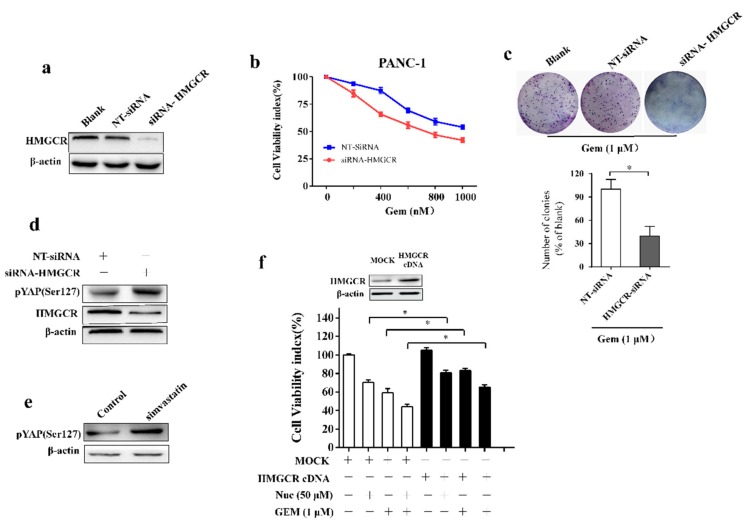
Downregulation of HMGCR increases the susceptibilities of PANC-1 cells to gemcitabine. (**a**) The protein level of HMGCR in PANC-1 cells transfected with siRNA-HMGCR. (**b**) Cell viability of cells transfected with siRNA-HMGCR in the presence of gemcitabine. (**c**) Clonogenicity of cells transfected with siRNA-HMGCR was determined after treated with gemcitabine for 24 h. (**d**) Western blot of HMGCR and pYAP(Ser127) from PANC-1 cells transfected with NT-siRNA and siRNA-HMGCR. (**e**) The protein levels of pYAP (Ser127) in PANC-1 cells incubated with HMGCR inhibitor simvastatin (20 μM) for 24 h. (**f**) Cells transfected with HMGCR overexpressed plasmid were treated with nuciferine and/or gemcitabine for 72 h and cell viability was determined. Data were showed as mean ± SD. Nuc, nuciferine; Gem, gemcitabine. * *p* < 0.05.

To evaluate the role of HMGCR on PC cells resistant to gemcitabine, PANC-1 cells were transfected with siRNA against HMGCR. Knockdown efficiency was confirmed by western blot (Figure 5a). Results showed that proliferative ability was lower in cells transfected with HMGCR siRNA compared with nonspecific RNA groups after gemcitabine treatment (Figure 5b). Furthermore, HMGCR siRNA enhanced the gemcitabine inhibition effect on colony-formation ability (Figure 5c). To gain mechanistic insight into HMGCR-mediated regulation of gemcitabine efficiency, we tested whether YAP is a substrate of HMGCR. Knockdown of HMGCR increased phosphorylation of YAP Ser 127 (Figure 5d). In line with this consistent, treatment with HMGCR inhibitor simvastatin inhibits YAP by phosphorylation Ser 127 in PC cells (Figure 5e). These events indicate that inhibiting HMGCR could restrain YAP by phosphorylation Ser 127, and therefore enhance the efficiency of gemcitabine in PC cells. As expected, overexpression of HMGCR reduced the growth inhibition caused by nuciferine and/or gemcitabine treatment in PC cells (Figure 5f). We propose that nuciferine-mediated HMGCR downregulation increases sensitivity of PC cells to gemcitabine.

### 3.6. Nuciferine Enhances PANC-1 Cells Sensitivity to Gemcitabine in Vivo

**Figure 6:**
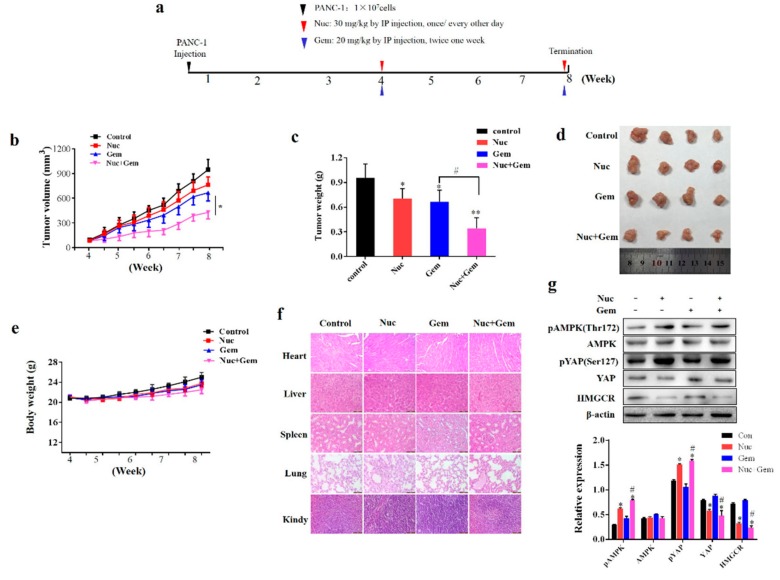
Nuciferine enhances gemcitabine efficiency *in vivo*. (**a**) PANC-1 cells were injected into mice (n = 4 mice per group) on day 0, and nuciferine or gemcitabine was administered as indicated. (**b**) Tumor volume was measured on the indicated time points. (**c**,**d**) At the end of the experiment, pancreatic tumor tissues were excised, photographed, and weighted. (**e**) Body weights of the mice were monitored over the treatment period. (**f**) HE staining of the major organs, Scale bars: 100 μm. (**g**) Western blot analysis on the expression of AMPK, pAMPK(Thr172), YAP, pYAP(Ser127) and HMGCR from respective tumor tissue lysates and relative density was determined. Nuc, nuciferine, Gem, gemcitabine. * *p* < 0.05 or ** *p* < 0.01 compared with control and # *p* < 0.05 compared with Gem group.

Finally, we evaluated whether nuciferine attenuates gemcitabine resistance of PC *in vivo*. We generated a subcutaneous xenograft model of PANC-1 cells in immunodeficient mice and then treated them with nuciferine and/or gemcitabine for 4 weeks (Figure 6a). Administration of nuciferine showed a similar effect as gemcitabine, with a remarkable reduction of the xenograft tumor volume and tumor weight compared with the control mice (Figure 6b–d), while combined treatment with nuciferine and gemcitabine further inhibited tumor growth (Figure 6b–d). Importantly, nuciferine and/or gemcitabine treatment for 4 weeks was well tolerated, without significant weight loss (Figure 6e). Histopathological analyses of vital organs (heart, liver, spleen, lung and kidney) also revealed that nuciferine and/or gemcitabine treatment didn’t result in the toxicity (Figure 6f).

We then detected the expression of pAMPK(Thr172)/HMGCR/pYAP(Ser127) in the xenograft tumors and found that combined nuciferine and gemcitabine treatment up-regulated the expression of pAMPK(Thr172) and pYAP(Ser127), while down-regulated levels of HMGCR compared with gemcitabine treatment (Figure 6g). Taken together, these data support our *in vitro* findings that nuciferine enhance the PC cells chemosensitivity to gemcitabine by inducing pYAP(Ser127) through AMPK-mediated downregulation of HMGCR.

## 4. Discussion

PC chemotherapy is frequently impeded by drug resistance. Recent work indicates that YAP activation induces PC cell chemoresistance and suggests that “switching off” of the Hippo-YAP pathway could help to prevent resistance to PC therapies [32]. The present study verified that treatment with nuciferine effectively attenuates gemcitabine resistance of PC via inhibition of YAP activity. Then, a key question arises, as to the mechanism for nuciferine-mediated inhibition of YAP.

AMPK is an energy sensor and master regulator of metabolism and AMPK activation occurs when the intracellular ATP/ADP ratio decreases. In our study, we found that nuciferine activated AMPK by decreasing cellular ATP/ADP levels in PC cells. Activated AMPK suppresses ATP consumption processes by inhibiting de novo synthesis of glucose, protein, and lipids and diverts cellular metabolism into energy-producing. In view of the essential role of cellular energy and metabolites for survival and growth, YAP is inhibited when energy level is low. Indeed, activation of AMPK induces an increase in YAP phosphorylation, which favors YAP localization to the cytoplasm, and finally decreases expression of YAP target genes [33]. Our research revealed that nuciferine induced YAP Ser127 phosphorylation and cytoplasmic retention and inhibited its transcriptional activity. However, cellular energy stress can inhibit YAP by both AMPK-dependent and independent mechanisms. We used siRNAs targeting AMPKa1 and AMPKa2 to confirm the mechanism of nuciferine-regulated YAP inhibition. AMPK knockdown greatly attenuated YAP Ser127 phosphorylation induced by nuciferine. Moreover, we observed that AMPK knockdown rescued the effects of nuciferine on YAP nuclear localization. These data indicate that AMPK is required to mediate YAP regulation in response to nuciferine treatment. A critical question then arises regarding the mechanism by which AMPK-mediated YAP inhibition occurs during nuciferine treatment.

Previous work indicated that AMPK directly binds to YAP and phosphorylates it. However, nuciferine did not enhance the combination of AMPK protein and YAP. Concordantly, nuciferine treatment did not increase YAP S61 or S94 phosphorylation. These findings argue against the possibility that nuciferine-mediated phosphorylation of YAP through AMPK directly binding to YAP. In response to unfavourable growth conditions, Lats1/2 is phosphorylated and activated, which phosphorylate and inhibit YAP. AMOTL1 could bind LATS1/2 and promote its kinase activity and YAP phosphorylation. De Ran et al. reported that AMPK can stabilize AMOTL1 to stimulate LATS activity, thereby promoting the phosphorylation of YAP on Ser 127 [19]. Our data indicated that nuciferine failed to decrease AMOTL1 protein levels and knockdown of LATS1/2 did not attenuate nuciferine-mediated YAP phosphorylation. These results arguing strongly against the possibility that nuciferine phosphorylates YAP protein through AMPK-mediated LATS1/2-dependent manner.

AMPK stimulation leads to the inhibition of HMGCR [34], which is the major regulators of the mevalonate pathway. Mevalonate metabolism are required for RHO function, which in turn is required for YAP activity [16,35,36]. Moreover, RHO regulates YAP is largely independent of the LATS1/2 Hippo pathway kinases. In our studies, we found that nuciferine decreased HMGCR protein levels and overexpression of HMGCR attenuated nuciferine-mediated YAP Ser 127 phosphorylation. Such findings are accordant with the studies which indicated that inhibition the mevalonate pathway with HMGCR inhibitor causes inhibition of YAP activity [37].

Increasing evidence has revealed that YAP plays a crucial role in regulating PC chemoresistance to gemcitabine [32,38]. We indicated that progressive decrease of pYAP(Ser127) is critical for YAP-mediated gemcitabine resistance. Moreover, our results showed that knockdown of HMGCR enhances the sensitivity of gemcitabine in PC cells, which is consistent with previous report that HMGCR inhibitor simvastatin increased the efficacy of gemcitabine in PC cells [39]. Thus, HMGCR inhibitor-mediated gemcitabine sensitivity is likely related to YAP Ser127 site phosphorylation. In support of this notion, inhibition of HMGCR increased pYAP(Ser127) in PANC-1 cells. Remarkably, overexpression of HMGCR rescued nuciferine-mediated gemcitabine sensitivity in PC cells. Thus, downregulation of HMGCR might serve as an important role for nuciferine to enhance gemcitabine efficiency by promoting pYAP(Ser127).

## 5. Conclusions

**Figure 7:**
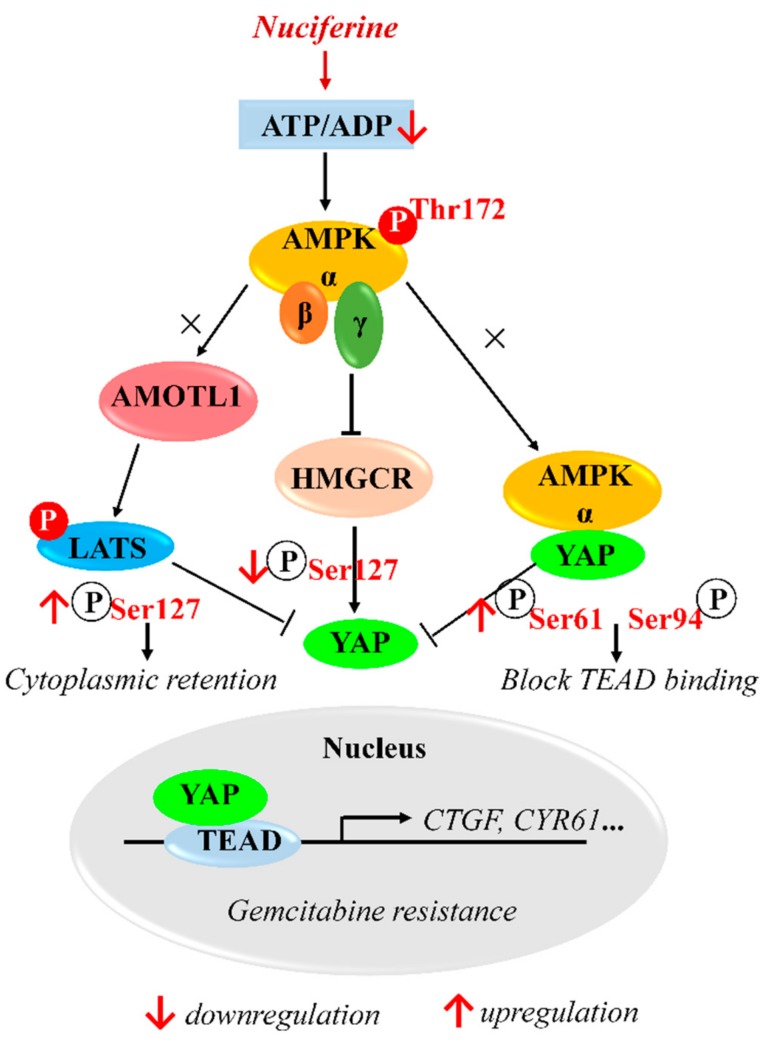
Schematic illustration of the mechanism of nuciferine to sensitize pancreatic cancer cells to gemcitabine.

The current study demonstrates that progressive decrease of pYAP(Ser127) is critical for YAP-mediated gemcitabine resistance. Nuciferine could attenuate gemcitabine resistance occurs in association with increase of pYAP(Ser127) through activation AMPK-mediated HMGCR downregulation in PC cells (Figure 7). This finding reveals a previously unrecognised role of nuciferine in antitumor action, via inhibition of YAP.
